# D-Lactic Acid as a Metabolite: Toxicology, Diagnosis, and Detection

**DOI:** 10.1155/2020/3419034

**Published:** 2020-06-17

**Authors:** Miroslav Pohanka

**Affiliations:** Faculty of Military Health Sciences, University of Defense, Trebesska 1575, Hradec Kralove CZ-50001, Czech Republic

## Abstract

Two enantiomers of lactic acid exist. While L-lactic acid is a common compound of human metabolism, D-lactic acid is produced by some strains of microorganism or by some less relevant metabolic pathways. While L-lactic acid is an endogenous compound, D-lactic acid is a harmful enantiomer. Exposure to D-lactic acid can happen by various ways including contaminated food and beverages and by microbiota during some pathological states like short bowel syndrome. The exposure to D-lactic acid cannot be diagnosed because the common analytical methods are not suitable for distinguishing between the two enantiomers. In this review, pathways for D-lactic acid, pathological processes, and diagnostical and analytical methods are introduced followed by figures and tables. The current literature is summarized and discussed.

## 1. Introduction

Toxicity of optical isomers is a specific issue that is not easy to be studied. When a compound is existing in a form of optical isomer (enantiomer), frequently one isomer is harmless or even necessary for homeostasis (e.g., L-amino acids) while the other isomer is toxic and able to interfere crucial pathways in the organism. On the other hand, some isomers do not exert significant problems (e.g., sugars). The toxicity depending on chirality is known from natural toxins, drugs, and pesticides [1–5]. Production of enantiopure drugs is a specific issue where high demands on quality of manufacturing and control are given. The manufacturing processes should be able to prefer or be fully selective to only one isomer. It can be reached by biocatalysis [6], advanced organic synthesis [7], and enantiospecific separation [8].

Though an unequal biological effect of optical isomers is known for a relatively long time, it is frequently underestimated because elaborative and expensive laboratory tests are necessary to distinguish the isomers. This review is focused on D-lactic acid which is, comparing to its L-isomer counterpart, problematic for the humans, and excessive intake can have fatal consequences. A survey of the actual literature, discussion of known facts, and ongoing research are provided in this work.

## 2. Comparison of the D- and L-Lactic Acids

**Figure 1:**
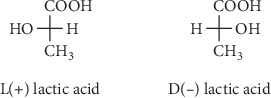
D- and L-lactic acids.

Lactic acid exists in the form of two enantiomers: D-lactic acid and L-lactic acid. In proper chemical terms, the L-lactic acid should be entitled L(+) lactic acid or S(+) lactic acid while the D variant is named D(-) lactic acid respective R(-) lactic acid. The isomers are depicted in Figure 1.

Lactic acid exists as a conjugated base lactate (L-lactate in the case of L-lactic acid and D-lactate in the case of D-lactic acid) in physiological pH 7.4, but the occurrence of conjugated base has no effect on chirality that is kept in the basic anion. L-Lactic acid is the natural enantiomer in humans and other higher forms of life. A normal level of L-lactate in human blood is in a range from 0.5 to 1 mmol/l, the increased level above the normal physiological range is called hyperlactatemia, and it can be initiated by some pathological processes. The low level of L-lactate is entitled as hypolactatemia [9]; however, hypolactatemia is quite a rare phenomenon comparing to indication of pathological states by hyperlactatemia which is a relevant finding in clinical biochemistry. Lactic acidosis occurs when the L-lactate concentration exceeds 4 mmol/l in plasma, and blood pH can drop under 7.35 in this situation. Hyperlactatemia in a mild form or a form of lactic acidosis can appear from several reasons like sepsis, hemorrhagic shock, cardiac arrest, trauma, poisonings, ischemia, burns, diabetic ketoacidosis, some types of cancer, and intense muscle activity [10–18].

**Figure 2:**
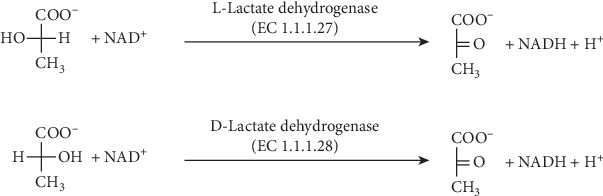
Oxidation of L-lactate by L-lactate dehydrogenase and D-lactate by D-lactate dehydrogenase to pyruvate.

Standard metabolism of L-lactate in most organisms is mediated by L-lactate dehydrogenase (EC 1.1.1.27) in the presence of NAD^+^ as a cofactor. The reaction is in its principle the same like the oxidation of D-lactate by D-lactate dehydrogenase (EC 1.1.1.28). The enzyme is quite evolutionary conserved because it can be found in eukaryotes, bacteria, and even archaea. Pyruvate and NADH are products of the reaction. The principle of L-lactate oxidation to pyruvate by L-lactate dehydrogenase is shown in Figure 2. The L-lactate dehydrogenase is involved in a basic metabolism tightly linked to glycolysis and gluconeogenesis, and it is a crucial part of the Cori cycle in humans and higher animals, but it also participates in fermentation processes [19–23].

Comparing to L-lactic acid, the D-lactic acid is not involved in basic metabolic processes of most life forms. Enzymes responsible for lactate metabolism including L-lactate dehydrogenase exert specificity to the L-isomer only, and they are not able to convert D-lactic acid. Only some exceptional L-lactate dehydrogenases like the enzyme from *Leuconostoc mesenteroides* are able to be involved in D-lactate conversion besides standard metabolism of L-lactate [24].

## 3. Basic Biochemical Pathways for D-Lactic Acid

Various bacterial species are able to produce D-lactate or both D- and L-lactates contemporarily. Many of them are involved in fermentation processes including the processes known from biotechnology industry. The genus of bacteria *Lactobacillus* produces D, L, and racemic mixture, genus *Pediococcus* produces either pure L or some strain racemic mixture, *Leuconostoc* and *Oenococcus* are producers of D-isomer, and genus *Weissella* produces either D-isomer or racemic mixture [25]. Engineered strains of bacteria can serve for optical isomer production. Aso and coworkers described *Lactococcus lactis*-based biotechnology for D-lactic acid production [26].

There are more pathways on how D-lactate is produced by microorganisms, fungi, and others. D-Lactate dehydrogenase (EC 1.1.1.28) is one of them. Fungi *Phytophthora undulata*, *Pythium debaryanum*, and *Sapromyces elongatus* [27] and bacteria *Lactobacillus delbrueckii* [28], *Lactobacillus bulgaricus* [29], *Escherichia coli*, *Fusobacterium nucleatum*, and *Pseudomonas aeruginosa* [30] are organisms known for expression of D-lactate dehydrogenase. D-Lactate dehydrogenase is an enzyme that converts D-lactate to pyruvate by contemporary reduction of NAD^+^ to NADH.

D-Lactate dehydrogenase (cytochrome) is another enzyme converting D-lactate. It is an enzyme known under code EC 1.1.2.4. It consumes D-lactate and needs two molecules of ferricytochrome c, while pyruvate and two molecules of ferrocytochrome c are reaction products. Activity of this enzyme was described for instance in plant *Arabidopsis thaliana* [31], yeast *Hansenula polymorpha* [32], and yeast *Saccharomyces cerevisiae* [33]. Participation of D-lactate dehydrogenase (cytochrome) in human metabolism has not been extensively researched. In a study, activity of the enzyme in human mitochondria and its deletion in individuals with deficient enzyme was described and the relation with appearance of D-lactate in urine of the individuals with deficient enzyme was discussed [34].

D-Lactate dehydrogenase (cytochrome c-553) is an enzyme similar to the D-lactate dehydrogenase (cytochrome) or probably in some cases the same biological structure. It is an enzyme oxidizing D-lactate to pyruvate with contemporary reduction of two molecules of ferricytochrome c-553 to two molecules of ferrocytochrome c-553 in a single step. This enzyme was described in bacterium *Desulfovibrio vulgaris* [35]. Detailed information about the enzyme are not reported in the current literature.

Glyoxalase 3 is an enzyme accepting D-lactate as a substrate and involved in its detoxification. The enzyme is also known under the name D-lactate dehydratase and code EC 4.2.1.130. It catalyzes reaction where D-lactate produces methylglyoxal (2-oxopropanal) and one molecule of water. This enzyme was identified in humans [36]. Glyoxalases 1 and 2 are enzymes involved in D-lactate conversion respective production in humans as well [37, 38]. Just as the system of glyoxalases makes a link between D-lactate and methylglyoxal, the methylglyoxal is further involved in forming advanced glycation end products, and in a wider look, it has a role in oxidative stress neurodegenerative disorders [39–42]. Enzymes involved in the lactate metabolism are summarized in Table 1.

## 4. Poisoning by D-Lactate and D-Lactic Acidosis

Production of lactic acids is common in biotechnologies of food processing, and manufacturing some types of food and beverages is typically based on or involves lactic acid production. Processes based on so-called lactic acid bacteria can be exampled [43]. Milk and milk beverage production can be complicated by D-lactic acid contamination depending on the type of bacterial presence [44]. There is a questionable effect of probiotics on the elevated level of D-lactate in blood circulation. Though some works point out potential risks of probiotics, the others show no evidence of increased D-lactate due to the probiotics and the final conclusion has not been done yet [45–48]. Moreover, production of D-lactate by probiotics is not related to all strains and use of D-lactic acid-producing strains is regulated, especially in products for children [25, 49]. In other experiments, the protective effect of probiotics was proven and their application was associated with reduction of D-lactate level [50].

Production of D-lactate by microbiota in the human organism is in limited amount under normal circumstances. D-Lactate can be overproduced by microbiota under specific circumstances like short bowel syndrome and jejunoileal bypass surgery further supported when a patient takes a meal with high sugar content [51, 52]. Abdominal compartment syndrome, a multiorgan failure associated with fluid accumulation within the peritoneal and retroperitoneal spaces, is another risk factor for increased D-lactate concentration in blood [53]. The presence of D-lactate can follow poisoning to heavy metals despite the exact mechanism remaining unknown. It was precisely described in a model of Wistar rats exposed to lead [54]. Infectious diseases and diseases with following inflammation processes can also cause or be in relation with increased D-lactate level [55–58]. D-Lactate can also serve as a marker of some infections and sepsis [59]. D-Lactic acidosis is formed due to overproduction of D-lactate [60]. D-Lactic acid can be contained in food and beverages prepared by biotechnology processes or contaminated by microorganisms or the other way; e.g., beer can be contaminated by D-lactic acid [61–64].

**Figure 3:**
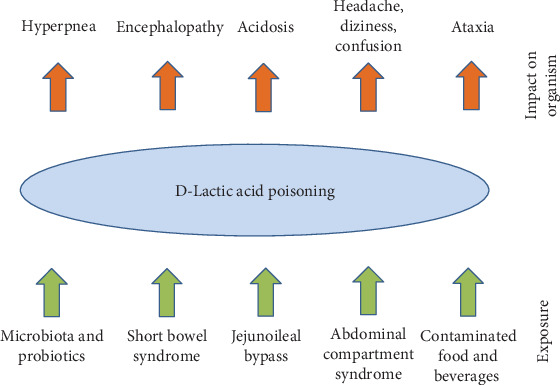
Overview of D-lactic acid exposures and impact on the organism.

D-Lactate initiates various pathological manifestations depending on the dose and individual conditions and specific metabolism. D-Lactate has a direct neurotoxic effect that is independent to the drop of blood pH and not common to L-lactate which was extensively scrutinized on Holstein calves as an experimental model [65]. In a case of D-lactate-poisoned lambs, acidosis, ataxic gait and preferred recumbency, and possible somnolence were identified and the symptoms can be suppressed by sodium bicarbonate [66]. Encephalopathy is also a common syndrome of D-lactate poisoning [67, 68]. The neurotoxic effect of D-lactate can be manifested by episodic confusion and hyperpnea as well [69]. Overall confusion, dizziness, headache, aggressive behavior, and memory loss are other symptoms following D-lactic acid poisoning [70]. An overview of types of D-lactic acid exposure and impact with manifestations is depicted in Figure 3. Though D-lactate can cause health complications, it is not a highly toxic compound because expected median lethal doses are quite high. The LD_50_ value level per orally poisoned rats is around 4.5 g/kg.

Because of environmental issues, good biocompatibility, and application in 3D printing, polylactic acid (synonym polylactide) becomes a highly preferred polymer that can be degraded in the nature [71–76]. The polylactic polymers in the current market are typically prepared from cheap racemic mixture of the D- and L-lactic acids, and it can be chemically named poly-DL-lactide. Use of optically pure isomers, from which poly-D-lactide and poly-L-lactide are formed, would make the final plastic products more expensive. On the other hand, the most commercially available lactic acid is the L enantiomer coming from the fermentation process, and the other significant source is a mixture fabricated chemically from acetaldehyde [77]. Hydrolysis of cellulose is possible as well [78, 79]; there are also protocols for production of D-lactate from methane [80]. Polylactic acid can be degraded by various processes, but hydrolysis of the ester bound is the most common reaction from the chemical point of view [81–83]; many organisms like bacterium *Rhodopseudomonas palustris* [84] and fungi *Aspergillus niger* and *Candida cylindracea* [85] are examples that have good ability to hydrolyze polylactic acid. Not a single enzyme is involved in polylactic acid hydrolysis. Lipases [85], carboxyl esterases [86], and serine proteases [87] were quoted as enzymes able to hydrolyze polylactic acid. Toxicity of polymers in the current market is generally low and hard to be measured. Some works however identified risks of the materials available in the current market, and the biodegradable materials like polylactic acid were surprisingly more harmful in the in vitro model [88]. Regarding polylactic acid, release of D-lactate due to metabolism should be taken into consideration; hence, products prepared from poly-DL-lactide and poly-D-lactide can be a certain risk when ingested.

## 5. Diagnosis and Therapy

Exposure to D-lactic acid can be diagnosed by standard biochemistry where some markers exert good dose-response relation. Besides this, the residual level of D-lactate in blood stream can be measured directly. The biochemical markers can point to a type of poisoning by an organic acid, but it is not easy to conclude that D-lactic acid is the causative agent of poisoning until further analytical tests are done. The exact assay of D-lactate in blood stream is not easy because L-lactate and D-lactate have the same physical and chemical properties, and most analytical methods are not able to distinguish them. At the same time, L-lactate will interfere in an assay because physiological concentration in blood or blood plasma is quite high; its concentration can reach 2.5 mmol/l, and it can even exceed 4 mmol/l under some conditions [89]. In the case of mild hyperlactatemia, L-lactate can reach 7 mmol/l, and 12 mmol/l in a moderate case, and patients with severe hyperlactatemia can have a concentration above 12 mmol/l [90].

Standard biochemical markers can help in the diagnosis of D-lactic exposure and estimate the presence of D-lactate in blood stream. D-Lactic acid quantitatively produces D-lactate under physiological conditions which results in the drop of blood pH with possible occurrence of high anion gap and metabolic acidosis. The impact of D-lactate on biochemical markers can be shown on a case of a 14-year-old boy that suffered from short bowel syndrome [69]. The patient had significantly higher concentration of D-lactate than L-lactate in blood plasma. While L-lactate was presented in a concentration of 2.89 mmol/l, D-lactate reached 11.2 mmol/l at the same time. Base excess was equal to -19.1 mmol/l, anionic gap to 28.2 mmol/l, and blood pH dropping to 7.23. In another described short bowel syndrome case, D-lactate reached a concentration of 8.9 mmol/l in blood while L-lactate was equal to 1.4 mmol/l and pH dropped to 7.30 with concurrent base excess of -11.8 mmol/l in a nine-year-old boy [91]. A five-year-old girl with short bowel syndrome was described in a case report [92]. During hospitalization, she had the following values for blood serum: pH 7.16, bicarbonate 5.2 mmol/l, base excess -20.2 mmol/l, L-lactate 0.92 mmol/l, and D-lactate 8.19 mmol/l. In all the three previous case reports, relapses happened and acidosis appeared again despite medical supervision. A survey of biochemical parameters from the introduced case reports is given in Table 2.

The biochemical markers can be of course influenced by other pathologies as presented in the case report by Heireman and coworkers for a patient contemporarily suffering from urosepsis, uncontrolled type 2 diabetes mellitus, paracetamol overdosing, and gastric bypass surgery [93]. This combination led to impaired renal function, lowered consciousness, hyperventilation, diarrhea, vomiting, and development of high anion gap metabolic acidosis. Acidosis and the change of other biochemical markers can be initiated by other chemical substances in a similar way as described here for D-lactic acid. Ethylene glycol and methanol can be mentioned as organic compounds initiating development of acidosis because organic acids are formed from them by metabolism—glycolic acid, glyoxylic acid, and oxalic acid from ethylene glycol and formic acid from methanol [94–96].

Recognizing of the exact type of poison is not possible without further identification by a chemical analysis. There are suitable enzymatic assays, bioassays, and biosensors that take advantage of enzymes specific to only one isomer [33, 97–99]. An electrochemical biosensor with D-lactate dehydrogenase from archaea *Candidatus caldiarchaeum subterraneum* was constructed by Satomura and coworkers, and it was suitable to determine D-lactate concentration in a range 0.03–2.5 mmol/l [97]. D-Lactate oxidoreductase (cytochrome) from yeast *Saccharomyces cerevisiae* served for a construction of an amperometric biosensor with exerted full selectivity to D-lactate [98]. D-Lactate can be easily measured by a conventional spectral analysis when combined with recognition potency of a selective enzyme. D-Lactate dehydrogenase was chosen as a tool for measurement of D-lactate in plasma of laboratory rats [99]. In this work, the enzyme oxidized D-lactate to pyruvate with simultaneous reduction of NAD^+^ to NADH. The created NADH provided a fluorescence signal with excitation at 340 nm and emission at 491 nm.

Standard analytical instrumental methods like chromatography or mass spectrometry are not generally suitable to distinguish optical isomers; however, some improvements can be done to make them selective. Ding and coworkers derivatized the D- and L-lactic acids by L-menthol, and the following gas chromatography (GC) with mass spectrometry (MS) was suitable for their selective analysis [100]. In another work, (S)(+)-1-(2-pyrrolidinylmethyl)-pyrrolidine served for D- and L-lactic acid derivatization and ultraperformance liquid chromatography with mass spectrometry analyzed the product of derivatization [101]. High-performance liquid chromatography with a chiral column is another opportunity for the assay purpose. It was for instance performed by Henry and coworkers for determination of D-lactate in urine, and they combined the high-performance liquid chromatography (HPLC) with MS and employed the Astec Chirobiotic™ R chiral column to achieve selectivity for the isomers [102]. A survey of analytical methods for D-lactic acid is given in Table 3.

Because the drop of pH is the direct consequence of D-lactate poisoning, application of sodium bicarbonate or any other drug for acidosis-resolving purposes is recommended as first-choice therapy [103–106]. Alteration of microbiota composition like suppression of D-lactic acid-producing *Lactobacillus* strains by competitive probiotics is a prophylactic way leading to prevention of D-lactate exposition [50]. Standard dialysis is effective in the cases of D-lactate poisoning and can be considered at least in the cases of severe acidosis. In a case report by Anderson and coworkers, reduction of D-lactate serum level from 0.59 mmol/l to 0.22 mmol/l was found when continuous ambulatory peritoneal dialysis was applied overnight [107].

## 6. Conclusions

D-Lactic acid is not a highly toxic compound representing serious threats to human life. On the other hand, it is a significant marker and toxic metabolite that can cause health problems and complicate other pathologies. Poisoning cannot be easily diagnosed because elaborative analytical methods are necessary to exactly distinguish the two isomers. Unfortunately, research on the mechanism of how D-lactic acid is produced, how to prevent complications, and how to diagnose poisoning is limited. Further research in this field is necessary. Economic importance of D-lactic acid will grow because of production of biodegradable materials where D-lactic acid is also used.

## Figures and Tables

**Table 1:** Enzymes involved in D-lactate metabolism.

Name of enzyme	EC number	Substrates	Products	Producing organisms	References
D-Lactate dehydrogenase	EC 1.1.1.28	D-Lactate + NAD^+^	Pyruvate + NADH	Fungi *Phytophthora undulata*, *Pythium debaryanum*, and *Sapromyces elongatus*, bacteria *Lactobacillus delbrueckii*, *Lactobacillus bulgaricus*, *Escherichia coli*, *Fusobacterium nucleatum*, and *Pseudomonas aeruginosa*	[27–30]
D-Lactate dehydrogenase (cytochrome)	EC 1.1.2.4	D-Lactate + 2 ferricytochrome c	Pyruvate + 2 ferrocytochrome c + 2H^+^	Plant *Arabidopsis thaliana*, yeast *Hansenula polymorpha* and *Saccharomyces cerevisiae*	[31–33]
D-Lactate dehydrogenase (cytochrome c-553)	EC 1.1.2.5	D-Lactate + 2 ferricytochrome c-553	Pyruvate + 2 ferrocytochrome c-553 + 2H^+^	Bacterium *Desulfovibrio vulgaris*	[35]
D-Lactate dehydratase (also glyoxalase 3)	EC 4.2.1.130	D-Lactate	Methylglyoxal + H_2_O	*Homo sapiens*	[36]

**Table 2:** Biochemical parameters of blood in D-lactate exposure case reports.

Patient	D-Lactate level	L-Lactate level	pH	Base excess	References
14-year-old boy suffering from short bowel syndrome	11.2 mmol/l	2.89 mmol/l	7.23	-19.1 mmol/l	[69]
9-year-old boy suffering from short bowel syndrome	8.9 mmol/l	1.4 mmol/l	7.30	-11.8 mmol/l	[91]
5-year-old girl suffering from short bowel syndrome	8.19 mmol/l	0.92 mmol/l	7.16	-20.2 mmol/l	[92]

**Table 3:** Biochemical parameters of blood in D-lactate exposure case reports.

Assay type	Principle of selectivity to D-isomer over L-isomer	References
Electrochemical biosensor	Selectivity given by enzyme D-lactate dehydrogenase	[97]
Electrochemical (amperometric) biosensor	Selectivity given by enzyme D-lactate oxidoreductase (cytochrome)	[98]
Optical (fluorimetry) test based on reduction of NAD^+^ to NADH	Selectivity given by enzyme D-lactate dehydrogenase	[99]
GC MS	D- and L-lactic acid derivatization by L-menthol	[100]
Ultraperformance liquid chromatography-MS	D- and L-lactic acid derivatization by (S)(+)-1-(2-pyrrolidinylmethyl)-pyrrolidine	[101]
HPLC-MS	Selectivity of a chiral column	[102]
